# Effects of compound Chinese herbal microecological preparations on growth performance, meat quality and rumen microflora of Simmental cattle

**DOI:** 10.3389/fgene.2026.1931836

**Published:** 2026-09-15

**Authors:** Peng Wang, Zixin Liu, Aoyu Jiang, Tian Xia, Zhiyong Yan, Chuanshe Zhou

**Affiliations:** 1 State Key Laboratory of Forge Breeding-by-Design and Utilization, Institute of Subtropical Agriculture, Chinese Academy of Sciences, Changsha, Hunan, China; 2 Hunan Provincial Key Laboratory of Animal Nutritional Physiology and Metabolic Process, National Engineering Laboratory of Livestock and Poultry Breeding Pollution Control and Resource Utilization Technology, Institute of Subtropical Agriculture, Chinese Academy of Sciences, Changsha, Hunan, China; 3 Chinese Academy of Sciences, Beijing, China; 4 Yuelushan Laboratory, Changsha, Hunan, China

**Keywords:** Chinese herbal-microecological preparations, growth performance, immune performance, meat quality, rumen microflora

## Abstract

**Introduction:**

Growth promotion and immune regulation represent the core research focuses in beef cattle production. As a combined formulation, compound Chinese herbal-microecological preparations (CCHMP) integrate the merits of plant-derived additives and probiotics, exerting dual effects on growth enhancement and immune modulation. This experiment was conducted using Simmental cattle as the experimental animals to evaluate the effects of CCHMP on their growth performance, meat quality, serum biochemical indicators, and rumen microbial flora.

**Methods:**

In this trial,two groups were set up, including a control group (fed with a basal diet) and an experimental group (fed with the basal diet supplemented with 1% CCHMP on a dry matter basis), with a feeding trial period of 120 days.

**Results:**

The results demonstrated that compared with the control group, dry matter intake, average daily gain , crude fat digestibility, serum levels of immunoglobulin G, catalase, total antioxidant capacity, glutathione peroxidase and total protein, as well as the lightness of the longissimus dorsi muscle and the contents of aspartic acid, threonine, glutamic acid, alanine, valine, isoleucine, leucine, tyrosine, phenylalanine, lysine, arginine, proline and Total Amino Acids were significantly upregulated in the experimental group (P < 0.05). Serum total cholesterol, high-density lipoprotein cholesterol, low-density lipoprotein cholesterol, interleukin-6 and shear force of the longissimus dorsi muscle were extremely significantly decreased in the experimental group (P < 0.05). Additionally, serum immunoglobulin A, cooking loss rate of muscle and ruminal ammonia nitrogen concentration were significantly reduced in the experimental group (P < 0.05). At the genus level, the relative abundance of Xylanibacter was significantly enhanced (P < 0.05), while that of Rikenellaceae RC9 gut group was significantly decreased in the experimental group (P < 0.05).

**Discussion:**

Dietary supplementation with 1% CCHMP on a dry matter basis effectively improves growth performance, optimizes muscle amino acid profiles, enhances meat quality and modulates rumen microbial composition in Simmental cattle. It also strengthens host health by regulating serum biochemical parameters, boosting antioxidant capacity and mediating immune function. This study reveals the CCHMP-mediated phylogenetic rearrangement of rumen microbiota and its association with the improvement of host phenotypes, providing new insights into understanding nutrition-microbe-host interactions.

## 1 Introduction

The rising demand for high-yield and high-quality beef cattle in China has accelerated the scaling-up and intensification of cattle production. China’s beef output has increased by more than 3% per year over the past 4 years (Nong, 2025). Meanwhile, high-density rearing increases the risk of disease incidence and impairs the health status of beef cattle. Against this backdrop, the development of green, safe feed additives with immunomodulatory functions has become an inevitable requirement for promoting the sustainable development of the beef cattle industry (Zhang et al., 2024).

Chinese herbal preparations confer multiple beneficial effects, including meat quality improvement and immune function enhancement. Featuring multi-target regulatory properties, low residual levels, and a low propensity for antibiotic resistance development, they serve as ideal functional additives for regulating animal immune function (Gao, 2024). Previous studies have demonstrated that dietary supplementation with 0.2% Chinese herbal preparations markedly improved beef tenderness and flavor substance contents, thereby enhancing meat quality (Li et al., 2023a). Meanwhile, Yi (2025) confirmed that daily supplementation with 200 g of Chinese herbal preparations significantly increased serum albumin, globulin and immune indicators, thereby improving dairy cow health. However, single application of Chinese herbal preparations presents obvious limitations, failing to regulate rumen microbial community structure and optimize rumen fermentation patterns, thereby exerting limited improvement on ruminant growth performance.

Microecological preparations, as novel green biological feed additives, which can effectively optimizes rumen fermentation characteristics in ruminants. Previous study demonstrated that supplementation with microecological preparations in diet could significantly improve rumen fermentation efficiency and average daily gain of ruminant animals (Wu et al., 2022a). Relevant dairy cow experiments also verified that dietary supplementation with microecological preparations at 20 g/d effectively improves rumen fermentation and milk quality (Jiang et al., 2025). Nevertheless, Single microecological preparations have limited anti-inflammatory, antioxidant and anti-stress effects, with poor immunomodulatory performance. Previous studies have preliminarily confirmed the application potential of these two types of green additives in livestock and poultry production. Nevertheless, most available researches have mainly focused on the individual supplementation of either Chinese herbal preparations or microecological preparations, which still have their respective limitations. In contrast, Compound Chinese herbal-microecological preparations (CCHMP) integrate bioactive components from multiple traditional Chinese medicinal plants with compound probiotics, which exhibit more prominent superiority over single additives, as they can enhance anti-inflammatory and antioxidant capacities, improve gastrointestinal motility, and ultimately promote growth performance of animals (Jiang et al., 2025; Wu et al., 2022b; Zhang et al., 2022; Li, 2024). Wu et al., 2023 highlighted that dietary supplementation with 1.6% CCHMP significantly improved growth performance and serum biochemical parameters in black goats. However, owing to the complex chemical compositions of traditional Chinese herbal medicines and the variable adaptability of compound probiotic strains in different animal hosts, the efficacy and functional mechanisms of CCHMP cannot be universally extrapolated. Accordingly, systematic investigations under specific experimental conditions are essential to elucidate its practical application potential and underlying action mechanisms. As a high-quality dual-purpose breed for milk and meat, Simmental cattle are characterized by high dressing percentage, excellent meat production performance, strong roughage tolerance and favorable disease resistance, and serve as the dominant beef cattle breed in China (Zhang et al., 2025a; Wu et al., 2023).

At present, the combined regulatory effects of combined Chinese herbal components and microecological preparations on beef cattle fattening have not been systematically elucidated, which limits their scientific application in large-scale commercial beef cattle farming. Therefore, this study used Simmental cattle as the experimental animals to systematically evaluate the impacts of CCHMP on growth performance, serum biochemical markers, and immune function of Simmental cattle, with a particular focus on deciphering its modulatory effects on the phylogenetic composition and ecological assembly of the rumen microbial community using full-length 16 S rRNA sequencing. This approach allows us to explore the potential genetic and ecological basis of the host-microbe interplay underlying the observed phenotypic improvements.

## 2 Materials and methods

### 2.1 Experimental design and feeding management

All animal experimental procedures were approved by the Animal Ethics Committee of the Institute of Subtropical Agriculture, Chinese Academy of Sciences (ISA-2025–0,062) and conducted in strict accordance with animal welfare and ethical guidelines. This experiment was carried out from 4 September 2025, to 10 January 2026, at Hunan Fangcao Ecological Agricultural Technology Co., Ltd., Changsha, China. A total of 16 fourteen-month-old Simmental cattle with similar body weight (457.75 ± 23.99 kg) were randomly divided into two groups with eight replicates per group. The sample size was determined *a priori* using G*Power software (3.1.9.7). Average daily gain was designated as the primary outcome for sample size estimation. The parameters were derived from previous study (Ramsay et al., 2025). Specifically, we assumed a minimum meaningful difference of 0.20 kg/d in ADG between the treatment and control groups, with a pooled standard deviation of 0.25 kg/d. This resulted in an estimated effect size of 0.80. An independent-samples t-test was selected as the statistical model, with the significance level (α) set at 0.05 and the statistical power (1-β) set at 0.80. Under these parameters, the required sample size was calculated as eight animals per group, which was therefore adopted in the present experiment. All cattle were raised in individual stalls, with a 7-day adaptation period and a 120-day formal trial period. Treatment groups were as follows: Control (CON): fed basal diet formulated in accordance with the NRC (2016) standards. Experimental (EXP): received the same basal diet supplemented with 1% CCHMP (Minchu Biotechnology Inc., Hunan, China) on a dry matter basis daily. CCHMP used in this trial was prepared as follows: Non-moldy and impurity-free herbal raw materials were rinsed with clean water and drained, then separately crushed and sieved through an 80–100 mesh screen. The resulting herbal powders were weighed according to the experimental ratio and blended in a mixer for 15–25 min. The blended powder was dried in an oven at 60 °C–70 °C until the moisture content was ≤ 8%. After cooling, the compound probiotic powder was added and fully mixed under low temperature, followed by subpackaging and sealing to obtain the final CCHMP product. The nutritional composition of the basal diet and components of CCHMPs are presented in Table 1 and Table 2, respectively. Diets were fully mixed before feeding. Two groups were reared under identical conditions following routine farm management. Feeding was performed at 08:00 and 15:00 daily, with residual feed controlled within 5% of the daily supply. All cattle had free access to drinking water. The barn was cleaned daily, bedding was replaced every 2 days, and the feeding and defecation status of cattle were monitored and recorded daily. Slaughtering and sample collection were conducted from days 116–120 of the formal trial.

**TABLE 1:** Composition and nutrient level of the experiment diets (air-dry basis).

Items	
Ingredients	Content (%)
Napier grass	40.96
Rice straw	25.86
Corn	21.57
Soybean meal	6.91
Wheat bran	2.21
Premix^1^	1.38
Brown sugar	0.55
Salt	0.28
NaHCO_3_	0.28
Total	100.0
Nutrition levels^2^	
Digestible energy/(MJ/kg)	9.34
Dry matter	38.05
Crude protein	14.91
Ether extract	2.81
Neutral detergent fiber	60.49
Acid detergent fiber	29.80
Crude ash	8.45
Ca	0.51
Total phosphorus	0.27

1 Formulated to provide (per kg of the basal diet): Fe 1,000–1,500 mg, Cu 200–500 mg, Zn 1,000–1,600 mg, Mn 900–1,600 mg, I 10–15 mg, vitamin A 120,000–350,000 IU, vitamin D_3_ 30,000–60,000 IU, vitamin E 350–700 mg, Se 10–15 mg, Co. 10–15 mg.

2 The gross energy, neutral detergent fiber, acid detergent fiber and crude protein are measured values, while the rest of the nutritional components was calculated according to NRC (2007).

**TABLE 2:** Composition of Chinese herbal compound microecological preparation.

Items	​
Ingredients^1^	Content (%)
Microecological preparation^2^	40.0
Hawthorn	30.0
Medicated leaven	10.0
Malt	10.0
Areca nut	5.0
Dried tangerine peel	5.0
Total	100.0

1 The herbal components (hawthorn, medicated leaven, malt, areca nut, and dried tangerine peel) were sourced from a single batch to ensure compositional consistency and conformed to the quality specifications of the Chinese Pharmacopoeia (2020 edition).

2 The compound direct-fed microbials (DFMs) were provided by Hunan Minchu Biotechnology Company Limited. The DFMs, consisted of Lentilactobacillus buchneri (L.buchneri) (2 × 108 CFU/g), Pediococcus pentosaceus (P.pentosaceus) (2 × 108 CFU/g), and Bifidobacterium longum (B.longum) (2 × 108 CFU/g). The three strains were identified at the species level by 16 S rRNA, gene sequencing and are deposited at the China General Microbiological Culture Collection Center (CGMCC) under accession numbers CGMCC, No. 11078 (L. buchneri), CGMCC, No. 31428 (P. pentosaceus), and CGMCC, No. 22949 (B. longum). Strain identification and safety evaluation were conducted in accordance with the Guidelines for the Identification and Safety Evaluation of Production Strains Used in Direct-Fed Microbials and Fermentation Products (Ministry of Agriculture and Rural Affairs of China, 2022). The DFMs, were supplied as a dry powder and stored in sealed, moisture-proof packaging at ambient temperature (below 25 °C) in a dry, well-ventilated area, protected from direct sunlight.

### 2.2 Sample collection and processing

Daily feed intake and residual feed were recorded throughout the formal trial to calculate dry matter intake (DMI). From day 116 of the experimental period, fecal samples were collected from each cattle via rectal sampling at fixed time points (10:00 and 17:00) after morning and evening feeding until the end of the trial, with a total collection amount of 300 g–500 g per individual. All fecal samples from each animal were thoroughly mixed, and 200 g subsamples were treated with 20 mL of 10% sulfuric acid for nitrogen fixation to prevent nitrogen loss, followed by immediate storage at −20 °C for subsequent crude protein (CP) determination. All feed and fecal samples were dried at 65 °C, ground, and passed through a 1 mm sieve for nutrient analysis. On day 120 of the trial, blood samples were collected from the caudal vein using vacuum tubes after the morning feeding (11:00). After standing at 4 °C for 2 h, samples were centrifuged at 2000 *g* for 15 min at 4 °C, and the separated serum was stored at −80 °C for serum biochemical measurement. After the morning feeding (11:00), rumen fluid was collected using a rumen catheter. To avoid sample contamination by saliva, the initial 50 mL of fluid was discarded during sampling. The obtained rumen fluid was transferred to 10 mL centrifuge tubes, centrifuged at 4,000 *g* for 15 min at 4 °C, and preserved at −80 °C to analyze rumen fermentation parameters and for 16 S rRNA gene sequencing. At the end of the trial, all cattle (eight per group) were slaughtered after 12 h of fasting and 3 h of water deprivation. Within 30 min post-slaughter, longissimus dorsi muscle samples were collected from the 12th to 13th ribs on the left side. After removing visible fat and connective tissue, each meat sample was cut vertically to the muscle fiber direction into five uniform blocks (3 cm × 3 cm×5 cm), vacuum-packaged, and stored at −20 °C for subsequent determination.

### 2.3 Sample analysis

#### 2.3.1 Growth performance measurement

On the early morning of day 1 of the formal trial, all cattle were weighed after overnight fasting to record the initial body weight. At the end of the trial, the final body weight was measured under the same fasting condition, and the average daily gain (ADG) was calculated accordingly.

$$ADG\;\left(kg/d\right)=\left(Final\;weight-Initial\;weight\right)\;/\;Experimental\;days$$

#### 2.3.2 Feed intake determination

The daily offered feed for each cow was accurately weighed and recorded before morning and evening feeding. Residual feed was collected and weighed 2 h after feeding. The average daily dry matter intake (DMI) of each individual was calculated according to the dry matter contents of the offered and residual feeds. The feed to gain ratio (F/G) was calculated based on the average daily DMI and ADG of each group, with the calculation formula presented as follows:

F/G=DMI / ADG

#### 2.3.3 Conventional nutrient composition analysis

The contents of dry matter (DM), crude protein (CP), and ether extract (EE) in diet and fecal samples were determined in accordance with the national standards GB/T 6435–2014, GB/T 6432–2018, and GB/T 6433–2006. Neutral detergent fiber (NDF) and acid detergent fiber (ADF) were measured using the Van Soest fiber analysis method. The calculation formulas for the apparent digestibility of each nutrient are presented as follows:

$$Apparent\;digestibility=100\times \left[1-\left(Ad*Nf\right)\;/\;\left(Af*Nd\right)\right]$$

Where *Ad* and *Af* are the acid-insoluble ash contents in diet and feces, respectively (g/kg); *Nd* and *Nf* are the contents of a certain nutrient in diet and feces, respectively (g/kg).

#### 2.3.4 Meat quality determination

Muscle pH at 45 min post-slaughter of the longissimus dorsi was measured using a portable pH meter (pH-STAR, Matthäus GmbH and Co. KG, Eckelsheim, Germany). Meat color parameters were determined with a colorimeter (CR-410, Wuxi Fosde Instrument and Equipment Company Limited, Jiangsu, China). Meat samples measuring 1 cm × 1 cm×1 cm were weighed accurately, sealed in cooking bags, and incubated in a constant-temperature water bath at 80 °C for 45 min. After cooling and surface drying, the samples were reweighed to calculate cooking loss. The treated meat samples were placed on a digital tenderness meter (C-LM3, Beijing Tianxiangfei Instrument Equipment Company Limited, Beijing, China) perpendicular to the muscle fiber direction, and sheared with a single blade under a 100 N force; the shear force values were recorded subsequently. Meat strips of 2 cm × 2 cm×4 cm were suspended at 4 °C for 48 h, followed by surface drying and weighing to determine drip loss. Briefly, 0.1 g of freeze-dried beef samples, ground and passed through a 40-mesh sieve, were mixed with 10 mL of 6 mol/L hydrochloric acid and hydrolyzed at 110 °C for 24 h. After constant volume adjustment, filtration, evaporation, and redissolution, the filtrate was transferred to sample vials. The contents of hydrolyzed amino acids were quantified using an automatic amino acid analyzer (L-8900, Hitachi, Ltd., Tokyo, Japan). The relevant calculation formulas are shown below:

$$Cooking\;loss=\left[\left(M1-M2\right)\;/\;M1\right]\times 100$$

Where: M1 = initial weight of beef before cooking (g); M2 = weight of beef after cooking (g).

$$Drip\;loss=\left[\left(W1-W2\right)\;/\;W1\right]\times 100$$

where: W1 = initial weight of beef before suspension (g); W2 = weight of beef after suspension (g).

#### 2.3.5 Rumen fermentation parameter analysis

Rumen fluid samples were centrifuged at 9,000 *g* for 10 min at 4 °C. Subsequently, 1.5 mL of the supernatant was mixed with 0.15 mL of 25% metaphosphoric acid for sample fixation to determine volatile fatty acids (VFAs). Microbial crude protein (MCP), ammonia nitrogen (NH_3_-N), and volatile fatty acids were measured according to the methods described by Wang et al. (2016), Weatherburn, (1967) and Zinn and Owens, (1986); respectively.

#### 2.3.6 Serum biochemical index detection

Serum biochemical indices, including immunoglobulin M (IgM), immunoglobulin A (IgA), immunoglobulin G (IgG), total protein (TP), albumin (ALB), alanine transaminase (ALT), aspartate transaminase (AST), alkaline phosphatase (ALP), blood urea nitrogen (BUN), glucose (GLU), triglyceride (TG), total cholesterol (T-CHO), lipase (LIP), non-esterified fatty acid (NEFA), and blood ammonia (BA), were determined using an automatic biochemical analyzer (Hitachi 7,600, Hitachi, Ltd., Tokyo, Japan). Commercial assay kits from Kelu Biotechnology Co., Ltd (Wuhan, China) and Mofan Biotechnology Co., Ltd (Nanjing, China) were used to measure serum total antioxidant capacity (T-AOC, Cat. No. AMHA2-M48), catalase (CAT, Cat. No. AMHA4-M48), malondialdehyde (MDA, Cat. No. AMHB6-M48), glutathione peroxidase (GSH-Px, Cat. No. ATHA2-M48), superoxide dismutase (SOD, Cat. No. AYHA7-M48), interleukin-2 (IL-2, Cat. No. ELK5645-48), interleukin-6 (IL-6, Cat. No. ELK2216-48), and tumor necrosis factor-α (TNF-α, Cat. No. ELK5925-48), in accordance with the manufacturer’s instructions.

#### 2.3.7 DNA sequencing and PCR amplification

Microbial DNA was extracted from rumen fluid samples of 16 Simmental cattle using the E. Z.N.A.® Soil DNA Kit (OMEGA Engineering, Inc., Connecticut, United States of America), following the manufacturer’s protocols. The V1-V9 variable regions of the bacterial 16 S rRNA gene were amplified by PCR using the primer pairs 27 F (5′-AGRGTTYGATYMTGGCTCAG-3′) and 1492 R (5′-RGYTACCTTGTTACGACTT-3′). The PCR procedure was as follows: initial denaturation at 95 °C for 2 min, followed by 27 cycles of denaturation at 95 °C for 30 s, annealing at 55 °C for 30 s, and extension at 72 °C for 60 s, with a final extension at 72 °C for 5 min. The 20 μL reaction system consisted of 4 μL of 5×FastPfu buffer, 2 μL of 2.5 mM dNTPs, 0.8 μL of each primer (5 μM), 0.4 μL of FastPfu polymerase, and 10 ng of template DNA, with three technical replicates for each sample. The amplified products were separated by 2% agarose gel electrophoresis and purified using an AxyPrep DNA Gel Extraction Kit (Axygen Biosciences, Union City, CA, United States of America) according to the manufacturer’s instructions. Qualified amplicons were ultimately sequenced on the PacBio Sequel II platform, and all amplicon sequencing services were completed by LingEn Biotechnology Co., Ltd (Shanghai, China). Raw sequences were subjected to quality filtering, splicing, and clustering to generate operational taxonomic units (OTUs). Rarefaction curves were plotted to evaluate sequencing depth, and alpha diversity was subsequently analyzed. Principal component analysis (PCA) was performed to assess beta diversity among samples. Linear discriminant analysis effect size (LEfSe) was then applied to identify differentially abundant microbial taxa between groups: the non-parametric Kruskal–Wallis test was first used to detect taxa with significant differences in relative abundance (*P* < 0.05), followed by linear discriminant analysis (LDA) to estimate effect sizes, with a threshold logarithmic LDA score of 2.0 set to define biologically relevant biomarkers. Finally, the microbial composition and relative abundance were statistically analyzed at the phylum and genus taxonomic levels.

#### 2.3.8 Data statistical analysis

The Shapiro-Wilk normality test of all data was conducted using SPSS 24.0 statistical software. All data conformed to a normal distribution. The analysis was performed using Student’s t-test using SPSS 24.0 statistical software. When *P* < 0.05, the data were statistically significant. There was no significant difference in the data when *P* > 0.05. When 0.05 ≤ *P* < 0.1, a trend was observed in the data. All data were presented as mean ± standard deviation.

## 3 Result

### 3.1 Effects of CCHMP on growth performance of simmental cattle

As shown in Table 3, compared with CON, the DMI and ADG of Simmental cattle in EXP were significantly increased, rising by 11.28% and 15.29%, respectively (*P* < 0.05). The final body weight marginally increased (0.05 ≤ *P* < 0.10).

**TABLE 3:** Effects of CCHMP on growth performance of Simmental cattle.

Items^1^	Treatments^2^		*P*-value
	CON	EXP	
Initial BW, kg	445.30 ± 8.52	463.20 ± 22.34	0.138
Final BW, kg	564.58 ± 13.92	600.72 ± 32.90	0.053
DMI, kg/d	9.31 ± 0.25	10.36 ± 0.60	0.009
ADG, kg/d	0.99 ± 0.05	1.15 ± 0.09	0.009
F/G	9.37 ± 0.21	9.05 ± 0.33	0.105

1 Initial BW, initial body weight, Final BW, final body weight; DMI, dry matter intake; ADG, average daily gain, F/G = Feed to Gain ratio. A P-value >0.05 indicates no significant difference between groups.

2 CON: control group, N = 8, basal diet; EXP: experiment group, N = 8, basal diet supplemented with 1% CCHMP, on a dry matter basis.

### 3.2 Effects of CCHMP on apparent nutrient digestibility in simmental cattle

As show in Table 4, compared with CON, the apparent digestibility of ether extract in EXP was significantly increased, with an increase of 40.56% (*P* < 0.05).

**TABLE 4:** Effects of CCHMP on nutrient apparent digestibility in Simmental cattle.

Items^1^	Treatments^2^		*P*-value
	CON	EXP	
DM, %	62.34 ± 3.85	62.63 ± 4.93	0.760
NDF, %	44.16 ± 3.95	41.58 ± 1.05	0.222
ADF, %	39.35 ± 4.04	41.37 ± 1.27	0.336
EE, %	62.37 ± 9.37	84.37 ± 8.74	0.005
CP, %	70.69 ± 1.46	71.93 ± 1.34	0.174

1 DM, dry matter; NDF, neutral detergent fiber; ADF, acid detergent fiber; EE, ether extract; CP, Crude Protein. A P-value >0.05 indicates no significant difference between groups.

2 CON: control group, N = 8, basal diet; EXP: experiment group, N = 8, basal diet supplemented with 1% CCHMP, on a dry matter basis.

### 3.3 Effects of CCHMP on serum biochemical, immune and antioxidant indices of simmental cattle

As shown in Tables 5 and 6, compared with CON, the serum concentrations of CHOL, HDL-C, LDL-C, IgA and IL-6 in Simmental cattle of EXP were significantly decreased, with respective reductions of 20.72%, 22.22%, 32.79%, 42.37% and 57.25% (*P* < 0.05); conversely, the level of IgG, CAT, T-AOC, GSH-PX, and TP were significantly increased (*P* < 0.05); In addition, serum IgM and SOD concentrations tended to increase marginally (0.05 ≤ *P* < 0.10).

**TABLE 5:** Effects of CCHMP on serum biochemical indices in Simmental cattle.

Items^1^	Treatments^2^		*P*-value
	CON	EXP	
TP, g/L	69.00 ± 4.61	74.98 ± 2.14	0.041
ALB, g/L	40.84 ± 2.13	41.30 ± 2.59	0.767
ALT, U/L	31.28 ± 6.93	29.96 ± 4.25	0.728
AST, U/L	66.00 ± 13.73	67.00 ± 5.24	0.885
ALP, U/L	149.20 ± 68.36	113.00 ± 14.95	0.306
BUN, mmol/L	3.94 ± 0.19	3.78 ± 0.18	0.214
GLU, mmol/L	1.90 ± 0.16	1.92 ± 0.68	0.952
TG, mmol/L	0.22 ± 0.04	0.18 ± 0.09	0.324
CHOL, mmol/L	2.51 ± 0.22	1.99 ± 0.18	0.003
HDL-C, mmol/L	1.80 ± 0.15	1.40 ± 0.16	0.003
LDL-C, mmol/L	0.61 ± 0.06	0.41 ± 0.06	0.001
LIPC, U/L	5.92 ± 0.51	6.56 ± 1.73	0.451
oNEFA, mmol/L	0.12 ± 0.03	0.12 ± 0.02	0.831
NH3L, umol/L	168.54 ± 17.97	153.30 ± 17.72	0.214

1 TP, total protein; ALB, albumin; ALT, alanine aminotransferase; AST, aspartate aminotransferase; ALP, alkaline phosphatase; BUN, blood urea nitrogen; GLU, glucose; TG, triglyceride; CHOL, cholesterol; HDL-C, High-Density Lipoprotein Cholesterol; LDL-C, Low-Density Lipoprotein Cholesterol; LIPC, hepatic lipase; oNEFA, Non-Esterified Fatty Acid, NH_3_L = Blood ammonia. A P-value >0.05 indicates no significant difference between groups.

2 CON: control group, N = 8, basal diet; EXP: experiment group, N = 8, basal diet supplemented with 1% CCHMP, on a dry matter basis.

**TABLE 6:** Effects of CCHMP on serum immune and antioxidant indices in Simmental cattle.

Items^1^	Treatments^2^		*P*-value
	CON	EXP	
Immunity			
IgM, mg/L	15.76 ± 8.89	30.10 ± 10.73	0.051
IgG, mg/L	111.13 ± 15.60	151.87 ± 30.44	0.038
IgA, mg/L	7.86 ± 2.41	4.53 ± 2.08	0.049
IL-2, pg/ml	118.83 ± 21.56	121.05 ± 25.55	0.899
IL-6, pg/ml	24.70 ± 8.25	10.56 ± 3.44	0.009
TNF-α, pg/ml	22.52 ± 14.86	15.04 ± 9.69	0.423
Antioxidant ability			
SOD, U/ml	102.11 ± 16.77	122.85 ± 10.56	0.060
CAT, U/ml	8.92 ± 0.75	10.09 ± 0.53	0.021
T-AOC, μmol/mL	0.31 ± 0.03	0.35 ± 0.02	0.023
GSH-PX, U/ml	143.76 ± 3.93	155.95 ± 3.20	0.015
MDA, nmol/ml	14.79 ± 3.15	13.81 ± 5.05	0.749

1 IgM = Immunoglobulin M, IgG = Immunoglobulin G, IgA = Immunoglobulin A, IL-2, Interleukin-2; IL-6, Interleukin-6, TNF-α, Tumor Necrosis Factor-α; SOD, superoxide dismutase; CAT = catalase, T-AOC, total antioxidant capacity; GSH-PX, glutathione peroxidase; MDA, Malondialdehyde. A P-value >0.05 indicates no significant difference between groups.

2 CON: control group, N = 8, basal diet; EXP: experiment group, N = 8, basal diet supplemented with 1% CCHMP, on a dry matter basis.

### 3.4 Effects of CCHMP on meat quality and hydrolyzed amino acid content in the longissimus dorsi of simmental cattle

As shown in Table 7, compared with CON, the shear force and cooking loss of the longissimus dorsi muscle in EXP were significantly decreased (*P* < 0.05), whereas muscle lightness (L*) was significantly increased (*P* < 0.05). Meanwhile, the crude ash content marginally decreased (0.05 ≤ *P* < 0.10).

**TABLE 7:** Effects of CCHMP on meat quality of Simmental cattle.

Items		Treatments^1^		*P*-value
		CON	EXP	
pH_45min_		6.26 ± 0.06	6.19 ± 0.15	0.310
Drip loss, %		24.93 ± 5.50	25.71 ± 8.39	0.867
Meat color	L*	34.11 ± 0.82	37.11 ± 4.25	0.037
	a*	22.07 ± 1.30	18.98 ± 4.65	0.196
	b*	9.72 ± 0.55	8.87 ± 2.06	0.396
Crude ash, %		4.89 ± 0.18	2.61 ± 0.72	0.065
Cooking loss, %		44.21 ± 1.56	42.07 ± 1.02	0.038
Shear force, N		126.58 ± 6.49	107.45 ± 10.01	<0.001

1 CON: control group, N = 8, basal diet; EXP: experiment group, N = 8, basal diet supplemented with 1% CCHMP, on a dry matter basis. A P-value >0.05 indicates no significant difference between groups.

As show in Table 8, compared with CON, the contents of aspartic acid (Asp), threonine (Thr), glutamic acid (Glu), alanine (Ala), valine (Val), isoleucine (Ile), leucine (Leu), tyrosine (Tyr), phenylalanine (Phe), lysine (Lys), arginine (Arg), proline (Pro) and TAA (Total Amino Acids) in the longissimus dorsi muscle of Simmental cattle in EXP increased by 15.22%, 15.56%, 14.19%, 16.95%, 16.67%, 17.78%, 15.85%, 11.43%, 15.79%, 17.98%, 17.74%, 22.22% and 15.95% (*P* < 0.05), respectively.

**TABLE 8:** Effects of CCHMP on the content of intermuscular hydrolyzed amino acids in Simmental cattle.

Items^1^	Treatments^2^		*P*-value
	CON	EXP	
Essential amino acids			
Asp, g/100 g	0.92 ± 0.04	1.06 ± 0.05	0.039
Lys, g/100 g	0.89 ± 0.04	1.05 ± 0.04	0.024
His, g/100 g	0.40 ± 0.01	0.46 ± 0.03	0.124
Gly, g/100 g	0.39 ± 0.02	0.50 ± 0.05	0.063
Leu, g/100 g	0.82 ± 0.04	0.95 ± 0.04	0.039
Met, g/100 g	0.23 ± 0.02	0.25 ± 0.01	0.204
Thr, g/100 g	0.45 ± 0.02	0.52 ± 0.02	0.040
Val, g/100 g	0.48 ± 0.02	0.56 ± 0.02	0.033
Ile, g/100 g	0.45 ± 0.02	0.53 ± 0.02	0.029
Phe, g/100 g	0.38 ± 0.02	0.44 ± 0.02	0.035
Non-essential amino acids			
Ala, g/100 g	0.59 ± 0.03	0.69 ± 0.04	0.043
Ser, g/100 g	0.39 ± 0.03	0.43 ± 0.03	0.206
Tyr, g/100 g	0.35 ± 0.01	0.39 ± 0.02	0.049
Glu, g/100 g	1.55 ± 0.11	1.77 ± 0.06	0.048
Arg, g/100 g	0.62 ± 0.03	0.73 ± 0.03	0.022
Pro, g/100 g	0.36 ± 0.03	0.44 ± 0.02	0.035
TAA, g/100 g	9.28 ± 0.44	10.76 ± 0.42	0.032

1 Asp = Aspartic acid, Lys = Lysine, His = Histidine, Gly = Glycine, Leu = Leucine, Met = Methionine, Thr = Threonine, Val = Valine, Ile = Isoleucine, Phe = Phenylalanine, Ala = Alanine, Ser = Serine, Tyr = Tyrosine, Glu = Glutamic acid, Arg = Arginine, Pro = Proline. A P-value >0.05 indicates no significant difference between groups.

2 CON: control group, N = 8, basal diet; EXP: experiment group, N = 8, basal diet supplemented with 1% CCHMP, on a dry matter basis.

### 3.5 Effects of CCHMP on rumen fermentation parameters in simmental cattle

As shown in Table 9, compared with CON, ruminal NH_3_-N concentration was significantly decreased in EXP, with a reduction of 46.81% over CON (*P* < 0.05).

**TABLE 9:** Effects of CCHMP on ruminal fermentation parameters in Simmental cattle.

Items^1^	Treatments^2^		*P*-value
	CON	EXP	
Acetate, mmol/L	71.08 ± 6.11	65.45 ± 7.68	0.238
Propionate, mmol/L	17.94 ± 0.95	17.84 ± 0.34	0.840
Isobutyrate, mmol/L	0.71 ± 0.16	0.74 ± 0.05	0.713
Butyrate, mmol/L	11.45 ± 0.91	11.1 ± 1.68	0.701
Isovalerate, mmol/L	1.07 ± 0.27	0.98 ± 0.11	0.529
Valerate, mmol/L	0.93 ± 0.10	0.86 ± 0.08	0.227
MCP, mg/ml	0.68 ± 0.37	0.69 ± 0.55	0.707
NH_3_-N, mg/dL	8.28 ± 0.62	5.64 ± 1.73	0.023
Acetate/Propionate	3.96 ± 0.16	3.79 ± 0.44	0.461

1 MCP, Microbial Crude Protein. A P-value >0.05 indicates no significant difference between groups.

2 CON: control group, N = 8, basal diet; EXP: experiment group, N = 8, basal diet supplemented with 1% CCHMP, on a dry matter basis.

### 3.6 Effects of CCHMP on rumen microflora in simmental cattle

#### 3.6.1 Analysis of rumen microbial α-diversity and β-diversity

The effects of the CCHMP on the α-diversity and β-diversity of the rumen microbial flora in Simmental cattle are shown in Figure 1. For α-diversity, the results in Figures 1a,b indicated that there were no significant differences in the Shannon index and Simpson index between EXP and CON (*P* > 0.05). In contrast, Chao1 index, ACE index, and PD_faith index exhibited a marginal decreasing trend (0.05 ≤ *P* < 0.10), suggesting that this preparation could reduce rumen microbial species richness and phylogenetic diversity to a certain extent. Principal Coordinates Analysis (PCoA) based on Bray-Curtis distance (Figures 1c,d) revealed that the first and second principal coordinates explained 52% and 13% of the total variation in microbial community structure, respectively, with a clear separation trend observed between the two groups. Permutational Multivariate Analysis of Variance further validated this trend, indicating that the difference in microbial community structure between the two groups approached statistical significance (*R* ^2^ = 0.54, *P* = 0.065). Collectively, these findings indicated that CCHMP could triggered a marginal differentiation between treatments.

**FIGURE 1:**
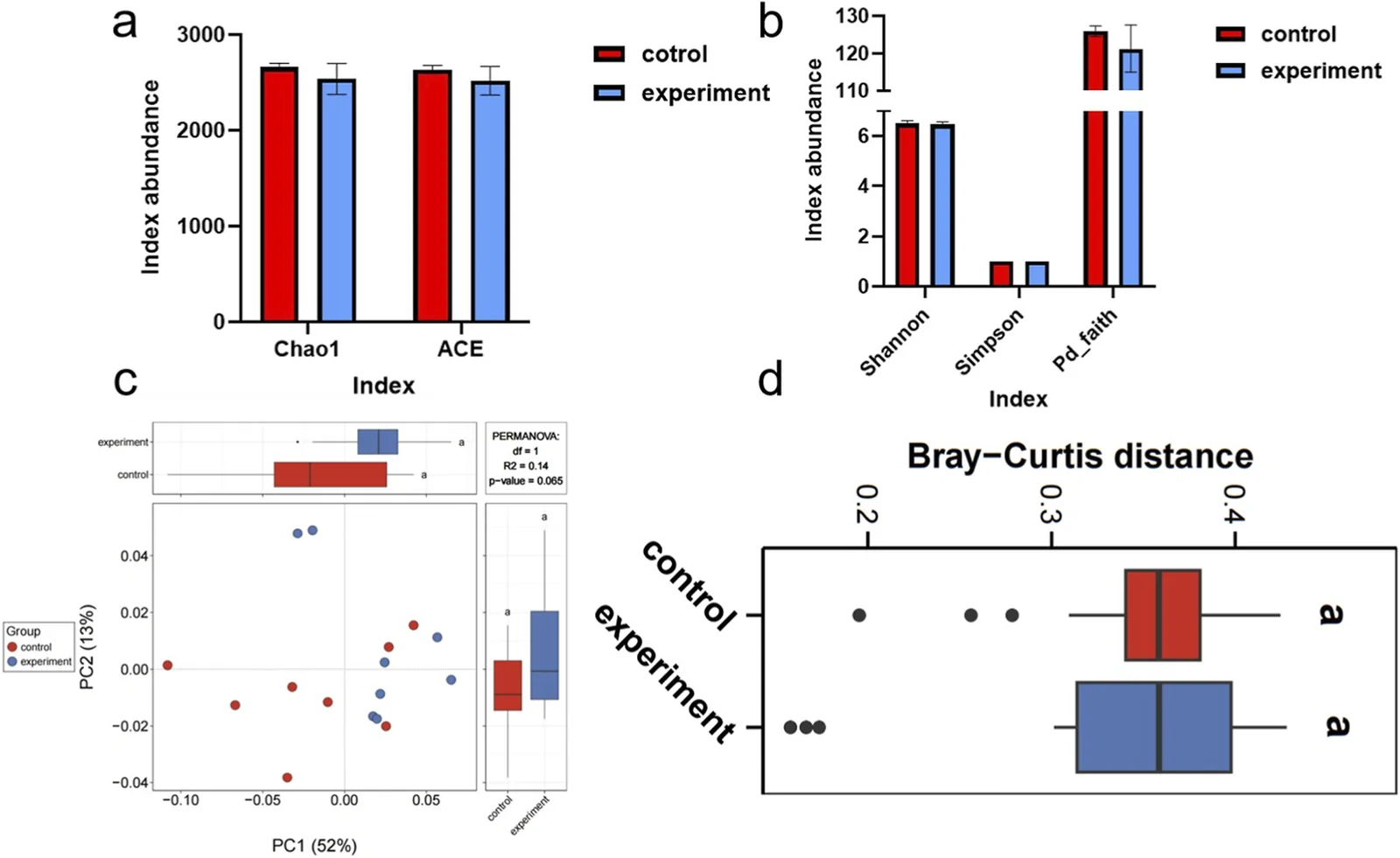
Effects of CCHMP on α and β-diversity of rumen microbial communities in Simmental cattle. **(a)** Analysis of ACE and Chao1 indices of rumen microbial communities in Simmental cattle; **(b)** Analysis of Shannon, Simpson and PD indices of rumen microbial communities in Simmental cattle; **(c)** Principal Coordinates Analysis (PCoA) of rumen microbial communities in Simmental cattle (with statistical tests); **(d)** Bray-Curtis distance boxplot of the rumen microbiota in Simmental cattle. Control: Control group, N = 8, basal diet; Experiment: Experiment group, N = 8, basal diet supplemented with 1% CCHMP on a dry matter basis.

#### 3.6.2 Analysis of rumen microbial community composition

At the phylum level, the top 10 rumen microbial taxa with the highest relative abundance in Simmental cattle across the two groups were selected for statistical analysis, as illustrated in Figure 2a. The top 10 predominant phyla included *Bacteroidota*, *Bacillota*, *Pseudomonadota*, *Spirochaetota*, *Fibrobacterota*, *Patescibacteria*, *Thermodesulfobacteriota*, *Actinomycetota*, *Verrucomicrobiota*, *Cyanobacteriota.* As presented in Figure 2b, relative to the control group, the experimental group showed a significant increase in the relative abundance of *Fibrobacterota* (*P* < 0.05); the relative abundance of *Verrucomicrobiota* exhibited a marginal increasing trend (0.05 ≤ *P* < 0.10).

**FIGURE 2:**
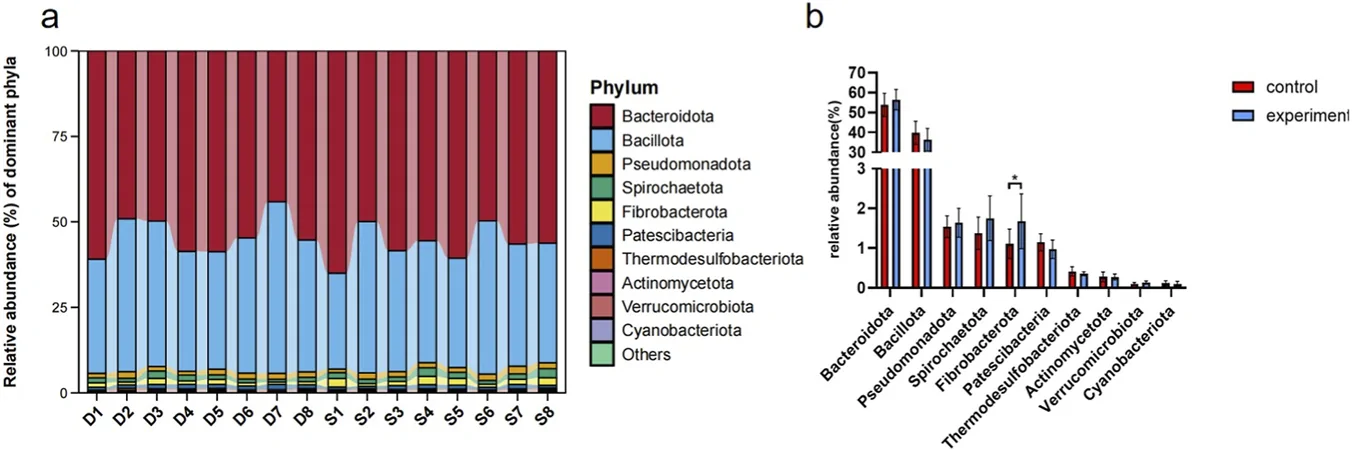
Phylogenetic composition of the rumen microbiota in Simmental cattle at the phylum level. **(a)** Composition of the rumen microbiota in Simmental cattle at the phylum level; **(b)** Effects of compound chinese herbal preparations on the ruminal microbiota at the phylum level in Simmental cattle. Data column without * indicate non-significant differences (*P* > 0.05), those with * indicate significant differences (*P* < 0.05), and those with ** indicate highly significant differences (*P* < 0.01). Control: Control group, N = 8, basal diet; Experiment: Experiment group, N = 8, basal diet supplemented with 1% CCHMP on a dry matter basis.

At the genus level, statistical analysis was performed on the top 10 rumen microbial taxa with the highest relative abundance in Simmental cattle from both groups, and the results are displayed in Figure 3a. The dominant genera ranking in the top 10 were *Xylanibacter*, Rikenellaceae *RC*9 *gut group*, Christensenellaceae *R-*7 *group*, *Paludibacter*, Prevotellaceae *UCG-*003, *Ruminococcus*, *Succiniclasticum*, *NK*4*A*214 *group*, *UCG-*001 and *UCG-*004. As illustrated in Figure 3b, relative to CON, EXP had a significantly higher relative abundance of *Xylanibacter* (*P* < 0.05), and the relative abundance of Prevotellaceae *UCG-*003 showed a marginal increase over CON (0.05 ≤ *P* < 0.10). Conversely, the relative abundance of Rikenellaceae *RC*9 *gut group* was significantly reduced (*P* < 0.05), and the relative abundance of *NK*4*A*214 *group* presented a marginal decrease over CON (0.05 ≤ *P* < 0.10).

**FIGURE 3:**
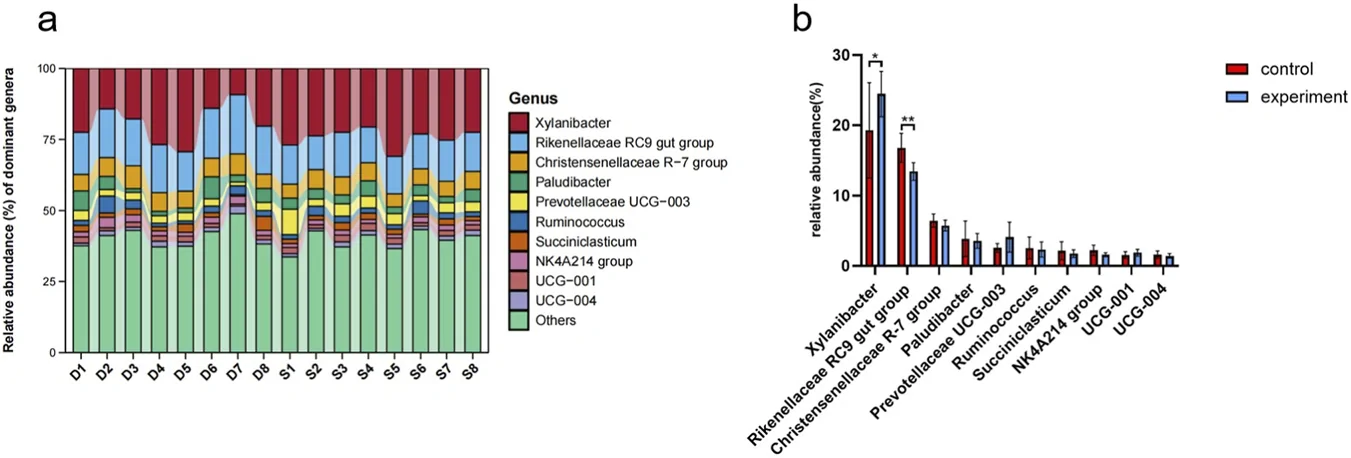
Phylogenetic composition of the rumen microbiota in Simmental cattle at the genus level. **(a)** Composition of the rumen microbiota in Simmental cattle at the genus level; **(b)** Effects of CCHMP on the ruminal microbial community at the genus level in Simmental cattle. Data column without * indicate non-significant differences (*P* > 0.05), those with * indicate significant differences (*P* < 0.05), and those with ** indicate highly significant differences (*P* < 0.01). Control: Control group, N = 8, basal diet; Experiment: Experiment group, N = 8, basal diet supplemented with 1% CCHMP on a dry matter basis.

Using an LDA threshold of >2, 62 differentially enriched taxa were identified, with nine in CON and 53 in EXP (Figures 4, 5). Taxonomic profiling showed that EXP was primarily enriched in *Spirochaetota*, *Fibrobacterota*, *Elusimicrobiota*, *Firmicutes*, and *Bacteroidota*, whereas CON was dominated by *Thermodesulfobacteriota* and *Lentisphaerota*.

**FIGURE 4:**
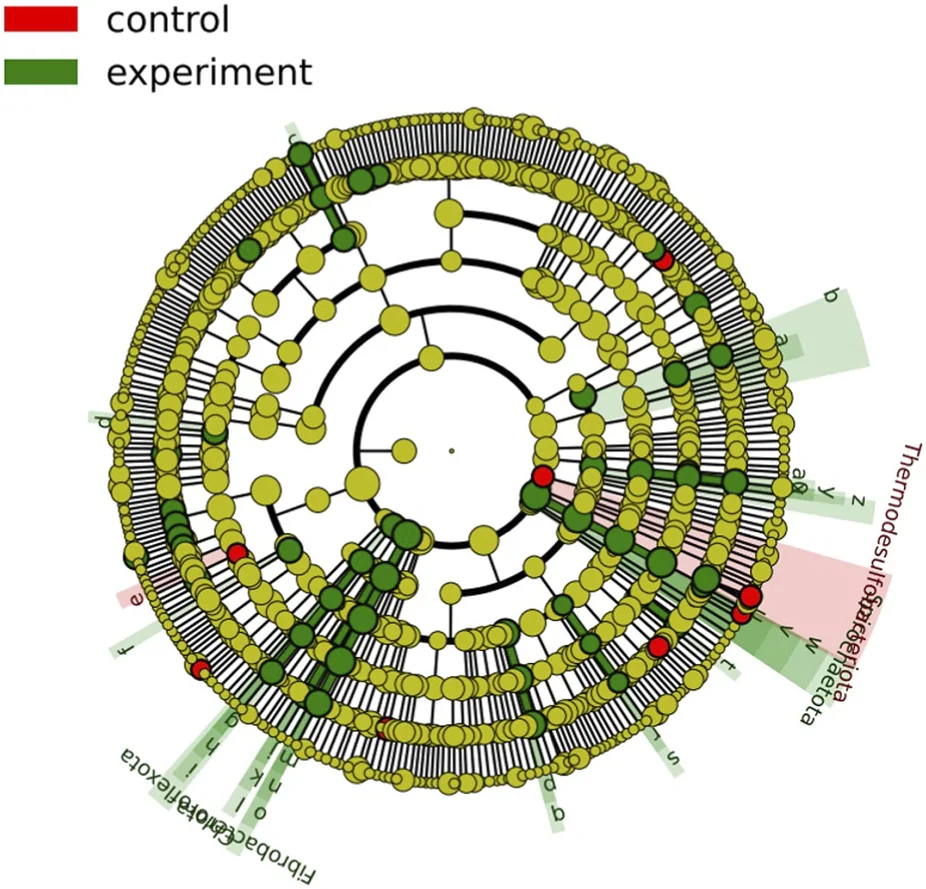
LEfSe circular cladogram analysis of rumen microbiota in Simmental cattle supplemented with CCHMP. Control: Control group, N = 8, basal diet; Experiment: Experiment group, N = 8, basal diet supplemented with 1% CCHMP on a dry matter basis.

**FIGURE 5:**
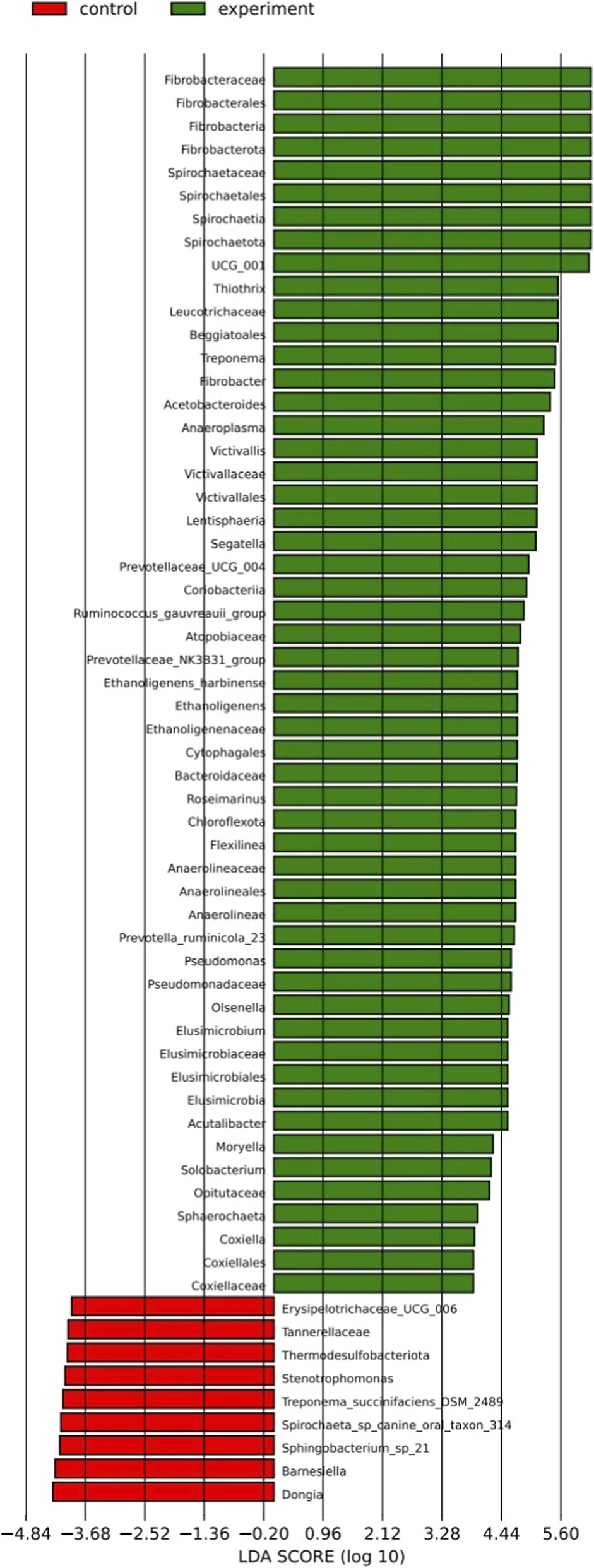
LEfSe analysis of rumen microbiota in Simmental cattle supplemented with CCHMP. The linear regression analysis (LDA) discriminant histogram counts the microbial groups that have significant effects on multiple groups. The larger the LDA score is, the greater the influence of the species abundance on the effect. The levels of taxonomy are phylum, family and genus; all-against-all was selected for multiple group comparison strategies, and LDA >2. Control: Control group, N = 8, basal diet; Experiment: Experiment group, N = 8, basal diet supplemented with 1% CCHMP on a dry matter basis.

#### 3.6.3 Correlation analysis

Physiological indicators with significant intergroup differences (including nutrient digestibility, meat quality, serum biochemical parameters, and rumen fermentation indices), were selected to conduct correlation analysis with the top 10 dominant rumen microbial genera at the genus level. The results are illustrated in Figure 6. Specifically, significant positive correlations were observed between Prevotellaceae *UCG-*003, LDL-C, *Xylanibacter* and IgG as well as the *NK*4*A*214 *group* and rumen NH_3_-N concentration (*P* < 0.05). An extremely significant positive correlation was detected between the Rikenellaceae *RC*9 *gut group* and rumen NH_3_-N concentration (*P* < 0.01). In contrast, *NK*4*A*214 *group* and Rikenellaceae *RC*9 *gut group* both exhibited extremely significant negative correlations with IgG, CHOL, HDL-C, and LDL-C (*P* < 0.01). Furthermore, the Rikenellaceae *RC*9 *gut group* was significantly negatively correlated with CAT activity and T-AOC capacity, while a significant negative correlation was found between the Christensenellaceae *R-*7 *group* and HDL-C content (*P* < 0.05).

**FIGURE 6:**
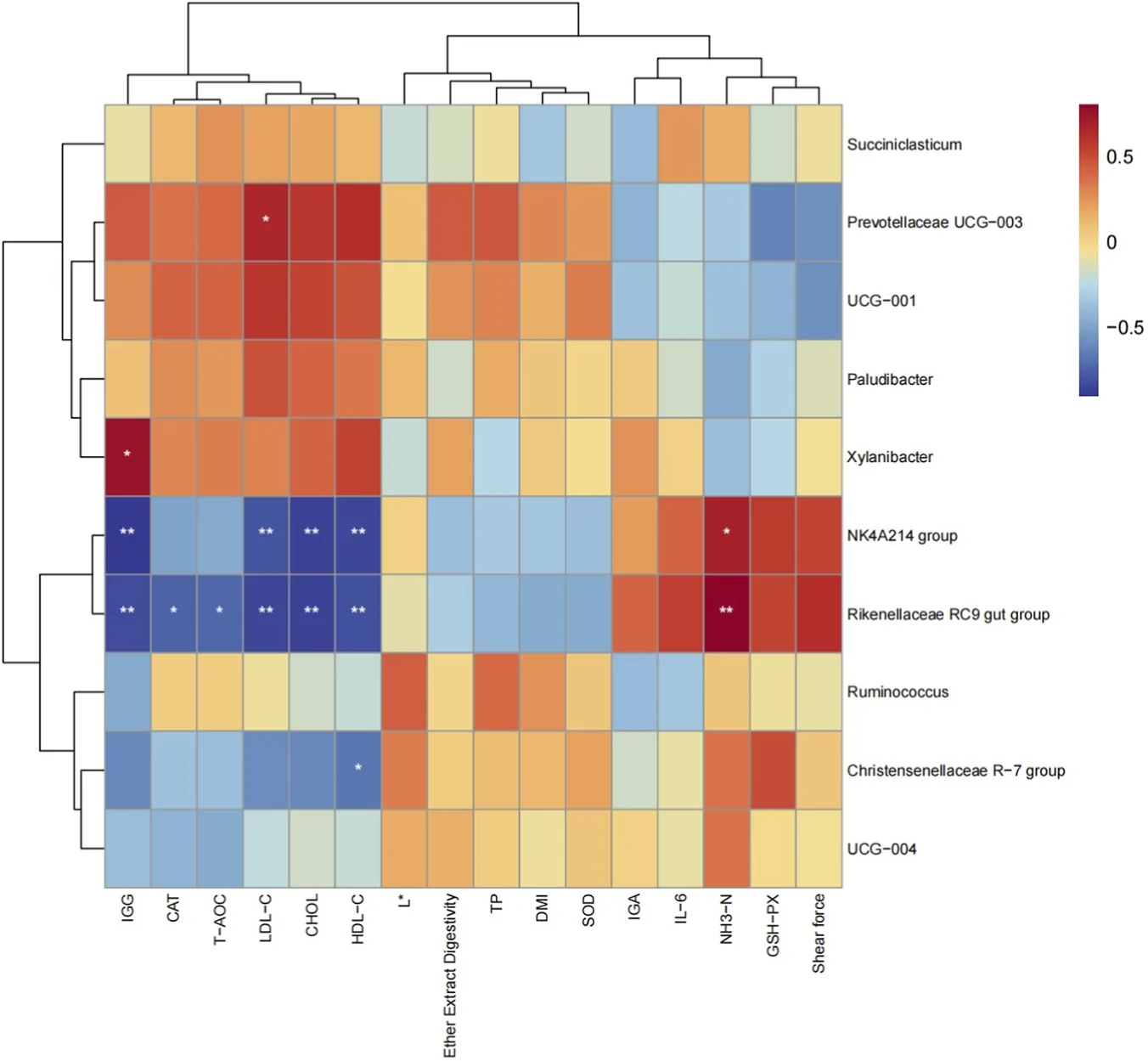
Correlation analysis between rumen microbiota and differential physiological indicators. The red block represents positive correlation, and the blue block represents negative correlation. Block without * indicate non-significant differences (P > 0.05),those with * indicate significant differences (P < 0.05), and those with ** indicate highly significant differences (P < 0.01).

## 4 Discussion

### 4.1 The effects of CCHMP on growth performance and nutrient digestibility of simmental cattle

Growth performance is a core indicator for evaluating the production value of beef cattle, and average daily gain (ADG) can directly reflect the growth status of livestock (Xu and Cai, 2023). Wu et al. (2022) reported that dietary supplementation with 1.6% CCHMP significantly increased ADG and DMI of black goats, which is consistent with the findings of the present study. The improved DMI observed in this trial may be attributed to the combined effects of multiple regulatory mechanisms. Firstly, the microecological components of CCHMP facilitate the enrichment of core fiber-degrading microbiota such as *Fibrobacterota* in rumen, thereby accelerating crude fiber degradation and rumen emptying rate, and further alleviating the inhibitory effect of rumen fill on feed intake (Zhang et al., 2025b; de Souza et al., 2017); Another potential mechanism involves the bioactive substances derived from hawthorn, malt and areca nut in CCHMP, such as hawthorn polysaccharides, malt polysaccharides and arecoline, can stimulate cholinergic M receptors and activate protein kinases. These bioactive substances enhance gastrointestinal peristalsis, invigorate the spleen and promote appetite, and ultimately elevate DMI (Wu et al., 2022b; Fu et al., 2022; Cherdthong et al., 2018). However, further studies are needed to elucidate the molecular mechanism by which CCHMP regulates digestive enzyme activity in the small intestine. Meanwhile, Previous research demonstrated that hawthorn extract could effectively enhance the activity of duodenal lipase, thereby promoting the crude fat digestibility in piglets, these findings are generally consistent with the present results (Fu et al., 2022). Therefore, the increased EE digestibility in Simmental cattle of EXP may be attributed to the hawthorn components in CCHMP, which enhance duodenal lipase activity and thereby facilitate the digestion and absorption of EE. However, as the active compound concentrations in the CCHMP were not quantified, this interpretation remains speculative and warrants further investigation.

Collectively, CCHMP significantly increases DMI in Simmental cattle through combined multi-mechanistic effects. Additionally, it improves the digestion and absorption efficiency of nutrients. The combination of these two beneficial effects ultimately contributes to a notable increase in the ADG of Simmental cattle.

#### 4.1.1 Effects of CCHMP on serum biochemical parameters of simmental cattle

Serum biochemical indices comprehensively reflect the health status, inflammatory levels, and nutritional metabolic status of animal organisms. TP is correlated with protein absorption and utilization, while CHOL, LDL-C and HDL-C serve as key markers of lipid metabolism (Zhu et al., 2025). Serum free amino acids serve as essential precursors for *in vivo* protein synthesis; their availability modulates hepatic protein anabolism, thereby regulating the circulating TP level (Kirsch et al., 1969). Bu et al. (2022) demonstrated that plasma amino acid concentrations in dairy cows are regulated by intestinal amino acid supply. Consistent with this, the increase in serum TP concentration in EXP may be related to the possibility that CCHMP accelerates rumen emptying, enabling more proteins and amino acids to reach the hindgut for subsequent digestion and absorption (Wu et al., 2022b; Fu et al., 2022; Cherdthong et al., 2018; Lu et al., 2017; Mariz et al., 2018). However, the underlying regulatory pathways remain to be exploration. The improvement of TP concentration can further optimize the steady state of amino acid transport in the body, enhance the efficiency of amino acid supply to muscle tissue, and ultimately promote an increase in amino acid deposition in muscles (McCormick and Webb, 1987). Meanwhile, Zheng et al. (2025) reported that hawthorn flavonoids could effectively decrease CHOL and LDL-C levels in serum and liver of hyperlipidemic mice, which is broadly consistent with the findings of the current study. Previous studies have demonstrated that cyanidin, a major flavonoid in hawthorn, can activate the AMPK signaling pathway in hepatocytes, thereby inhibiting the transcription and maturation of *SREBP-*1*c* (Tajima-Shirasaki et al., 2017; Gong et al., 2023; Fangfang et al., 2026), and ultimately reduce the serum concentrations of CHOL, HDL-C and LDL-C (Tajima-Shirasaki et al., 2017; Gong et al., 2023; Fangfang et al., 2026; Li et al., 2026). Extrapolating from these reports, it is plausible that similar bioactive compounds present in the hawthorn component of CCHMP may have contributed to the observed reduction in serum cholesterol-related indicators in EXP. However, this interpretation remains speculative and warrants further investigation.

The above results indicate that CCHMP can significantly improve the protein metabolism and lipid metabolism of Simmental cattle, effectively enhance the liver synthesis function and muscle amino acid deposition, and play an important role in maintaining the body health of Simmental cattle.

#### 4.1.2 Effects of CCHMP on serum immune indicators of simmental cattle

This study found that the CCHMP significantly reduced the serum levels of IL-6 and IgA in Simmental cattle of EXP. Previous studies have confirmed that protocatechuic acid, an active component in hawthorn, can target and inhibit the nuclear factor-kappa B (NF-κB) signaling pathway, block the transmission and activation of the inflammatory cascade, and downregulate the transcriptional secretion of IL-6 to alleviate inflammation (Zhang et al., 2022). Further studies have demonstrated that the expression of IgA-related genes is positively correlated with the degree of local chronic inflammation (Chen et al., 2026). Based on these observations, it is plausible that the decreases in serum IL-6 and IgA levels in Simmental cattle under the experimental conditions are at least partly related to bioactive compounds such as protocatechuic acid in the CCHMP, which targets and inhibits NF-κB to further alleviate the inflammatory response. In addition. Liu et al. (2023) found that Medicated Leaven could inhibit the expression of macrophage-inducible nitric oxide synthase (iNOS) and reduce the production of high-concentration nitric oxide (NO). As a potent negative regulatory factor for B lymphocyte activation, proliferation, and antibody secretion, the reduction in high-concentration NO has been shown to relieve its inhibitory effect on B lymphocytes, thereby promoting the secretion of IgG and IgM (Liew, 1995). Accordingly, the elevated serum IgG and IgM concentrations in Simmental cattle of EXP may be associated with the suppression of NO production, potentially mediated by Medicated Leaven.

The above results indicate that these findings demonstrate that CCHMP can effectively relieve inflammatory responses and strengthen humoral immunity, which is critical for maintaining the physiological health of Simmental cattle.

#### 4.1.3 Effects of CCHMP on serum antioxidant indicators of simmental cattle

The present study observed that CCHMP significantly increased serum GSH-Px and CAT activities as well as T-AOC levels, and induced a marginal rising in SOD activity in Simmental cattle. Tang et al. (2022) demonstrated that dietary supplementation with 600 mg/kg hawthorn extract significantly elevated serum SOD and GSH-Px activities in weaned piglets. Chen et al. (2025) also reported that 1% hawthorn powder supplementation markedly improved serum GSH-Px and CAT activities, as well as T-AOC levels, in broilers. These findings are consistent with the results of the present study. Hawthorn is known to contain abundant natural antioxidant bioactive compounds. Notably, vitexin-2-O-rhamnoside (VR) has been shown to promote the phosphorylation of the PI3K/Akt signaling pathway in immune cells, with reported effects including enhanced serum GSH-Px, CAT, SOD activities and T-AOC levels (Ni et al., 2022). Meanwhile, flavonoids derived from hawthorn leaves can inhibit the expression of *HMGCR* and *SCAP*, thereby elevating serum SOD and CAT activities in rats (Chen et al., 2025; Hu et al., 2022). Furthermore, soluble wheat bran xylan and yeast cell wall polysaccharides in Medicated Leaven have been found to effectively enhance serum GSH-Px and SOD activities in animals (Li et al., 2025). Extrapolating from these reports, it may be speculated that the elevation of serum GSH-Px and SOD activities in EXP of Simmental cattle could be partly related to the activation of the PI3K/Akt signaling pathway by VR and the downregulation of *HMGCR* and *SCAP* expression by hawthorn leaf flavonoids. Additionally, functional polysaccharides derived from Medicated Leaven may have contributed through their potential free radical scavenging activity.

Collectively, these findings suggest that CCHMP may enhance the antioxidant capacity of Simmental cattle, potentially through the modulation of molecular pathways and gene expression involved in redox homeostasis, thereby reducing intracellular free radical accumulation and mitigating oxidative damage. However, the specific molecular targets and underlying mechanisms remain to be elucidated.

### 4.2 Effects of CCHMP on meat quality and hydrolyzed amino acid content in longissimus dorsi of simmental cattle

Meat quality is a core economic indicator for evaluating the production performance of beef cattle. Cooking loss and shear force reflect muscle water-holding capacity and meat tenderness, respectively (Piórkowska et al., 2016). Meanwhile, muscle amino acid profile dominates the nutritional value and flavor quality of beef (Han et al., 2025). Li (2023) documented that dietary supplementation with 150 mg areca nut extract markedly increased muscle L* value and reduced shear force in broilers, which is consistent with the results of the present study. Epicatechin and syringic acid in areca nut ethanol extract, functional polysaccharides from Medicated Leaven, and flavonoids from hawthorn leaves all exhibit potent antioxidant activities (Chen et al., 2025; Hu et al., 2022; Li et al., 2025; Ji et al., 2022). Accumulating evidence suggests that the oxidation levels of muscle lipids and proteins are critical factors regulating muscle L*, shear force, and cooking loss (Elisabeth and Steven, 2005; Rowe et al., 2004; Elisabeth and Steven, 2005). Based on these observations, it is plausible that the increased L* and decreased cooking loss and shear force in the longissimus dorsi muscle of Simmental cattle in EXP may be associated with the inhibition of muscle lipid and protein oxidation potentially mediated by antioxidant active components in CCHMP. In addition, this study also observed that the contents of 12 intermuscular amino acids in the muscle tissue of Simmental cattle in EXP were significantly increased, including 4 flavor amino acids (Asp, Glu, Ala, Lys),3 antioxidant amino acids (Tyr, Arg, Glu), and 6 essential amino acids (Thr, Val, Ile, Leu, Phe, Lys). *L. buchneri*, *P. pentosaceus*, and *B. longum* can upregulate the activities of rumen nitrate reductase, glutamate dehydrogenase, and alanine dehydrogenase, optimize the body’s ammonia cycle, facilitate the assimilation of ammonia nitrogen for glutamate production, and mediate the synthesis of various amino acids as well as amino acid metabolic cycles via transamination (Jiang et al., 2025; Pengpeng and Tan, 2013). Meanwhile, the hawthorn polysaccharides, malt polysaccharides, and arecoline in CCHMP have been shown to promote gastrointestinal peristalsis, accelerate rumen emptying, which could increase the direct absorption of amino acids in the posterior intestine and improve the utilization rate of amino acids (Wu et al., 2022b; Fu et al., 2022; Cherdthong et al., 2018). Collectively, the probiotic modulation and bioactive constituent intervention may produce a combined effect that could improve amino acid absorption and metabolic homeostasis, elevates serum TP levels, and thereby contribute to significant increases in twelve amino acids in the longissimus dorsi muscle of Simmental cattle.

### 4.3 Effects of CCHMP on rumen fermentation parameters in simmental cattle

Results of the present study revealed that within the normal physiological fluctuation range, dietary supplementation with CCHMP markedly reduced ruminal NH_3_-N concentration in Simmental cattle. Previous studies have demonstrated that arecoline can stimulate cholinergic M receptors, activate protein kinases, and promote gastrointestinal peristalsis (Wang et al., 2023; Kaushal et al., 2010). Arecoline contained in CCHMP accelerates the ruminal NH_3_-N evacuation rate by promoting gastrointestinal peristalsis. Furthermore, previous studies have confirmed that the *NK*4*A*214 *group* and Rikenellaceae *RC*9 *gut group* are key functional bacteria responsible for ruminal protein and urea degradation (Li et al., 202b3; Yan et al., 2025). 16 S rRNA sequencing results revealed that dietary supplementation with CCHMP markedly reduced the relative abundances of these two bacterial groups in the rumen of Simmental cattle. Accordingly, it may be speculated that the suppression of ruminal nitrogen-degrading bacteria by CCHMP could partially account for the reduction in ruminal NH3-N content in EXP. However, this possibility needs to be confirmed by subsequent mechanistic experiments. Interestingly, ruminal MCP production was not decreased in Simmental cattle of EXP. Jiang et al. (2025) reported that CCHMP’s microecological components could enhance the activity of nitrate reductase and facilitate the assimilation and utilization of ammonia nitrogen by rumen bacteria, thereby providing sufficient substrates for MCP synthesis, which may offer a plausible explanation for the stable MCP concentration. Rumen emptying rate increasing facilitates the digestion and absorption of dietary protein and amino acids in the hindgut, potentially improving amino acid utilization efficiency at the source (Lu et al., 2017; Mariz et al., 2018). Meanwhile, CCHMP may help maintain normal ruminal MCP synthesis by modulating nitrate reductase activity, possibly preventing insufficient microbial protein supply associated with reduced NH_3_-N. Such dual regulatory effects potentially enhance the overall absorption and deposition efficiency of amino acids, and may thus contribute to the observed increases inserum total protein content and muscle amino acid levels.

The above results suggest that CCHMP may regulate ruminal NH_3_-N concentration while maintaining normal MCP synthesis, thereby potentially improving rumen health and contributing to muscle amino acid deposition in Simmental cattle.

### 4.4 Effects of CCHMP on ruminal microbiota in simmental cattle

Rumen microbes play a pivotal role in feed digestion, nutrient synthesis, maintenance of internal environmental homeostasis, and overall health of ruminants. The results of this study demonstrated that no significant differences were detected in ruminal microbial α-diversity across groups. Whereas the β-diversity presented evident community separation, the overall taxonomic profile was remodeled, with the relative abundances of fiber-degrading bacteria and predominant genera significantly increased. LEfSe analysis further identified key differential microbial taxa associated with CCHMP supplementation. A total of 53 taxa with an LDA score >2 were enriched in EXP, whereas only nine such taxa were enriched in CON. This finding suggests that CCHMP can moderately modulate the composition of the rumen microbial community and directionally enrich fiber-degrading functional flora, without significantly affecting the richness and diversity of rumen microbes (Wang et al., 2012; Mao et al., 2013).

At the phylum level, the dominant rumen bacterial phyla of Simmental cattle in EXP were *Bacteroidota*, *Bacillota* and *Pseudomonadota*. This community structure characteristic was basically consistent with the composition pattern of rumen microbial flora in ruminants fed mainly with roughages such as rice straw and napier grass (Zhang et al., 2026a). The experimental results indicated that the CCHMP significantly increased the relative abundance of *Fibrobacterota* in rumen. *Fibrobacterota* is a type of key extracellular polysaccharide and crude fiber-degrading flora, and previous studies have confirmed that *Fibrobacterota* plays a crucial role in the degradation of rice straw (Zhang et al., 2026b). These results indicate that CCHMP can effectively enhance the rumen’s ability to degrade and metabolize crude fiber substrates such as rice straw, while also in turn facilitating the decomposition and utilization of Chinese herbal polysaccharides by rumen microbes.

Genus-level analysis revealed distinct differences in rumen microbial community structure between treatments. *Xylanibacter*, Rikenellaceae *RC*9 *gut group* and Christensenellaceae *R-*7 *group* were identified as the dominant genera in the rumen of experimental cattle. Specifically, the relative abundance of *Xylanibacter* was significantly upregulated, while that of Rikenellaceae *RC*9 *gut group* was markedly decreased in EXP. Meanwhile, Prevotellaceae *UCG-*003 showed an increasing trend, whereas the *NK*4*A*214 group exhibited a declining trend. *Xylanibacter* can utilize various hemicelluloses, pectins in plant cell walls, and plant storage polysaccharides, serving as a core rumen microbial population responsible for the digestion of crude fiber and structural carbohydrates (Murovec and Accetto, 2024). Jiang et al. (2025) reported that *L. buchneri*, *P. pentosaceus* and *B. longum*could enhance the ecological competitive capacity of *Prevotella* in rumen. Furthermore, taxonomic studies have demonstrated that *Xylanibacter* and *Prevotella* share highly homologous gene sequences, some researchers suggest that the two genera may belong to the same taxonomic group (Sakamoto and Ohkuma, 2012). Accordingly, we speculate that the marked enrichment of *Xylanibacter* in EXP may be attributed to the modulation of *Prevotella* proliferation by microecological ingredients contained in CCHMP. Rikenellaceae *RC*9 *gut group* primarily ferments crude fiber as its main substrate (Shaopeng et al., 2025). Accordingly, the marked reduction in its relative abundance is presumed to result from nutritional substrate competition with *Xylanibacter*. The above inferences are speculative, and the underlying regulatory network needs to be explored in depth. Accumulating evidence has confirmed that Prevotellaceae *UCG-*003 is negatively associated with subacute ruminal acidosis and ruminal inflammatory levels (Liu et al., 2025). Collectively, these findings indicate that CCHMP exerts prominent effects on improving fiber degradation efficiency and rumen health by restructuring the rumen microecosystem of Simmental cattle.

From a phylogenetic perspective, the significant enrichment of *Fibrobacterota* and *Xylanibacter* suggests that CCHMP selects for specific bacterial lineages with a known genetic capacity for complex carbohydrate degradation (Zhang et al., 2026b; Murovec and Accetto, 2024). Simultaneously, the suppression of Rikenellaceae *RC*9 *gut group*, a lineage often associated with protein and urea degradation (Li et al., 2023b; Yan et al., 2025), indicates a niche shift in the rumen ecosystem. This community-wide phylogenetic restructuring, driven by CCHMP, aligns with the improved nitrogen utilization and enhanced antioxidant/immune status of the host, thereby providing indirect genetic clues for understanding how nutritional interventions may modulate host metabolism and immune responses by selecting for specific functional lineages. In this sense, the present study contributes a phylogenetic-genetic perspective to the field of nutritional ecology, suggesting that 16 S rRNA-based community analysis can generate testable hypotheses about the genetic potential of microbiota that may underpin host phenotypic responses to dietary interventions.

It is important to acknowledge, however, that while full-length 16 S rRNA gene sequencing provides high-resolution taxonomic and phylogenetic classification, it does not directly capture functional gene expression or metabolic activities. Therefore, our interpretations of microbial functionality are primarily inferred from taxonomic identities and existing literature on well-characterized genera. Future studies integrating metagenomics or metatranscriptomics are warranted to validate these phylogenetically inferred functional hypotheses and to elucidate the definitive molecular mechanisms.

### 4.5 Correlation analysis between rumen microbiota and phenotypic traits in simmental cattle

To explore the interactions between rumen microbiota and phenotypic traits, Spearman’s correlation analysis was performed in the present study. Previous studies have demonstrated that *NK*4*A*214 *group* serves as a functional rumen microbial group capable of efficiently degrading proteins and peptides (Li et al., 2023b). Rikenellaceae *RC*9 *gut group* harbors genes related to urea transport and degradation, enabling it to supplement rumen ammonia nitrogen via urea hydrolysis (Yan et al., 2025). Both genera can mediate ruminal ammonia nitrogen production via protein degradation and urea hydrolysis. It is therefore plausible that this mechanism largely accounts for the significant positive correlation between their abundance and ruminal ammonia nitrogen concentration.

Rikenellaceae *RC9 gut group* is a typical propionate-high-yielding microbial group (Conte et al., 2022). Propionate, in turn, can downregulate the activity of 3-hydroxy-3-methylglutaryl-coenzyme A (HMG-CoA) reductase, thereby inhibiting cholesterol production (Wright et al., 1990). *In vitro* experiments on rat hepatocytes have confirmed that propionate can effectively inhibit cholesterol synthesis in hepatocytes, which is basically consistent with the results of the correlation analysis (Demigné et al., 1995). It is possible that the higher abundance of Rikenellaceae *RC*9 *gut group* is associated with elevated propionate production, which in turn may suppress the activity of HMG-CoA reductase. This could partially explain the negative correlations between this microbial group and serum TC, HDL-C, LDL-C levels. Furthermore, as mentioned above, both Rikenellaceae *RC*9 *gut group* and *NK*4*A*214 *group* can modulate short-chain fatty acid synthesis, thereby coordinately regulating gastrointestinal immune competence and antioxidant capacity (Li et al., 2023b; Qingqing et al., 2026; Ma et al., 2012). A decrease in the abundance of both genera leads to increased gastrointestinal oxidative stress and inflammatory burden, this could in turn activates the nuclear factor erythroid 2-related factor 2 (Nrf2) and NF-κB signaling pathways. This activation might elicit a compensatory host response, characterized by the initiation of the antioxidant defense system and the activation of humoral immune responses, ultimately resulting in elevated serum antioxidant and immune indices (Helmut, 2015; Wang et al., 2020). To a certain extent, this finding elucidates the intrinsic mechanism behind the significant negative correlations of Rikenellaceae *RC*9 *gut group* and *NK*4*A*214 *group* with serum IgG, CAT, and T-AOC levels. However, the specific mechanism remains to be further verified.

Accumulating evidence has confirmed that *NK*4*A*214 *group* is a typical functional microbial group with butyrate-producing capacity (Li et al., 2023b). Du et al., 2020 demonstrated that butyrate can upregulate the expression of ATP-binding cassette transporter A1 (ABCA1), promote cholesterol efflux, and thereby reduce serum cholesterol levels in mice. Based on this, it can be hypothesized that *NK*4*A*214 *group* may regulate the expression of ABCA1 through butyrate secretion, thereby mediating the levels of serum TC, HDL-C and LDL-C. *Xylanibacter* is capable of degrading xylan to produce short-chain fatty acids. These metabolites can inhibit the activity of histone deacetylase (HDAC) and promote B cell differentiation as well as IgG secretion (Chang et al., 2014). This mechanism may underlie the significant positive correlation between *Xylanibacter* abundance and serum IgG levels.

The primary limitation of this study is its small sample size. Therefore, this study should be considered a preliminary exploratory investigation. The core value of its findings lies in the proposal of hypotheses rather than their verification; these hypotheses can provide research directions and candidate targets for subsequent confirmatory studies conducted in larger-scale cohorts.

This study demonstrates that dietary supplementation with CCHMP effectively improves ruminal fermentation characteristics and meat quality, and significantly increases average daily gain, immune function, and antioxidant capacity in Simmental cattle. These beneficial effects are mediated through the promotion of gastrointestinal peristalsis, activation of immune cells, and driving phylogenetic restructuring of the rumen microbial community. Phylogenetic analysis based on full-length 16 S rRNA sequencing revealed that CCHMP significantly enriched fiber-degrading bacterial lineages while suppressing ureolysis-associated lineages. These phylogenetic shifts in the microbial community provide clues, at the genetic information level, for understanding how nutritional interventions modulate host nitrogen metabolism, fiber digestion, and immune responses by selecting for specific functional lineages. However, these inferences warrant further validation through metatranscriptomic or host transcriptomic approaches. Notably, CCHMP exhibited a potential immunosuppressive effect, as evidenced by reduced IgA levels, suggesting a dual nature of its immunomodulatory role. This observation underscores the need for further in-depth investigations to optimize the formulation and application regimen of CCHMP. In summary, this study employs phylogenetic analysis of the rumen microbiota as an effective tool to bridge nutritional interventions with host phenotypic responses, thereby providing a foundational framework for future research on the genetic ecology of nutrition-microbiome-host interplay in ruminants.

## Data Availability

The original contributions presented in the study are publicly available. This data can be found here: https://doi.org/10.5281/zenodo.21337951.
